# A series of unfortunate events: Mind the windpipe

**DOI:** 10.1002/ccr3.3484

**Published:** 2020-11-24

**Authors:** Anam Miloud Elarabi, Mutaz Majdi Albakri, Mona Babiker, Mousa Hussein, Mushtaq Ahmed, Rashid Mazhar, Tasleem Raza

**Affiliations:** ^1^ Pulmonology Department Hamad General Hospital Hamad Medical Corporation Doha Qatar; ^2^ Thoracic Surgery Department Hamad General Hospital Hamad Medical Corporation Doha Qatar

**Keywords:** COVID‐19 pneumonia, prolonged intubation, surgical resection, tracheal stenosis

## Abstract

Since we started seeing post‐COVID pneumonia patients in our clinics, tracheo‐laryngeal stenosis should be kept in mind as an important sequela of prolonged intubation (>7 days) particularly in those who are persistently symptomatic.

## Graphical abstract

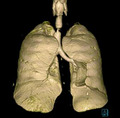

## 1 CASE DESCRIPTION

Tracheal stenosis can be a disastrous consequence of prolonged endotracheal intubation. In the COVID‐19 era, vigilance must be maintained not to overlook this complication.

**FIGURE 1:**
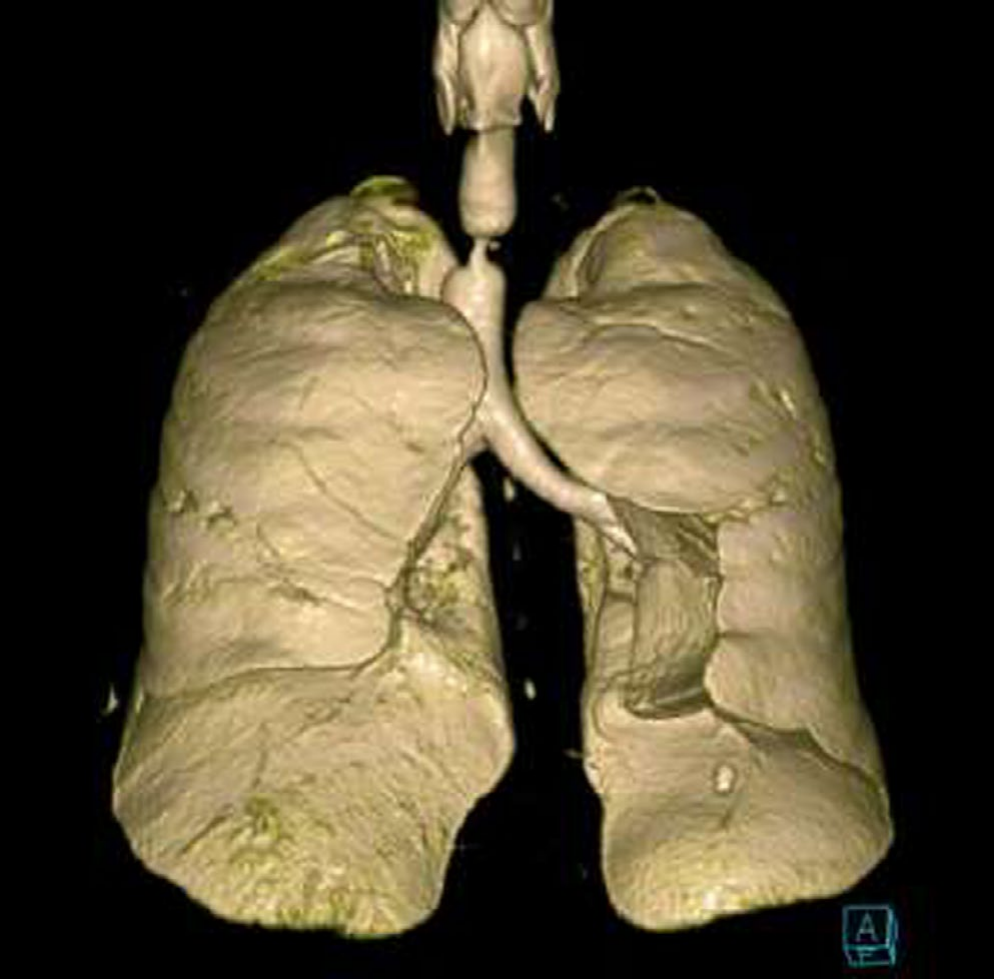
3D reconstruction of CT‐Chest showing the level and extent of the tracheal stenosis (anterior view)

**FIGURE 2:**
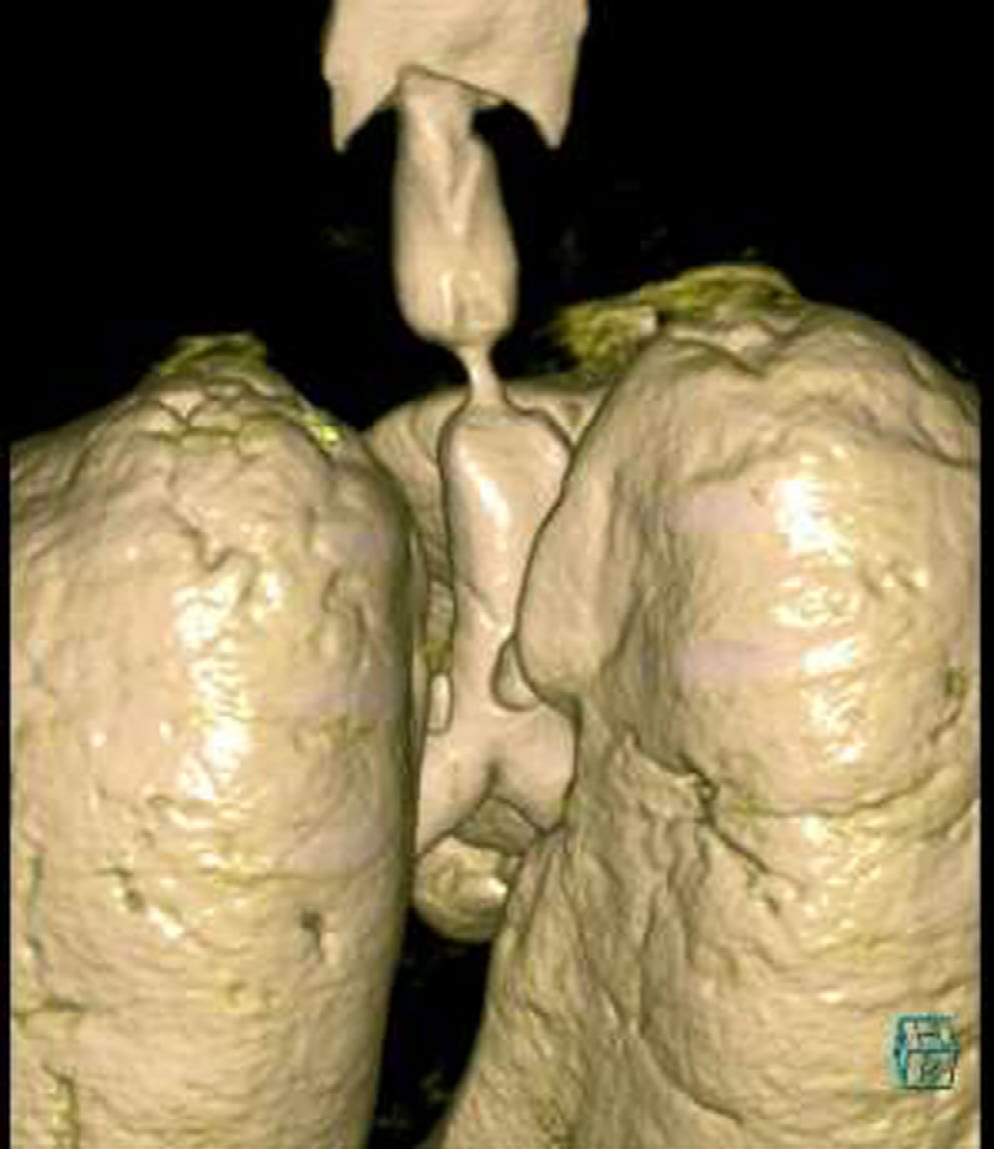
3D reconstruction of CT‐Chest showing the level and extent of the tracheal stenosis (posterior view)

**FIGURE 3:**
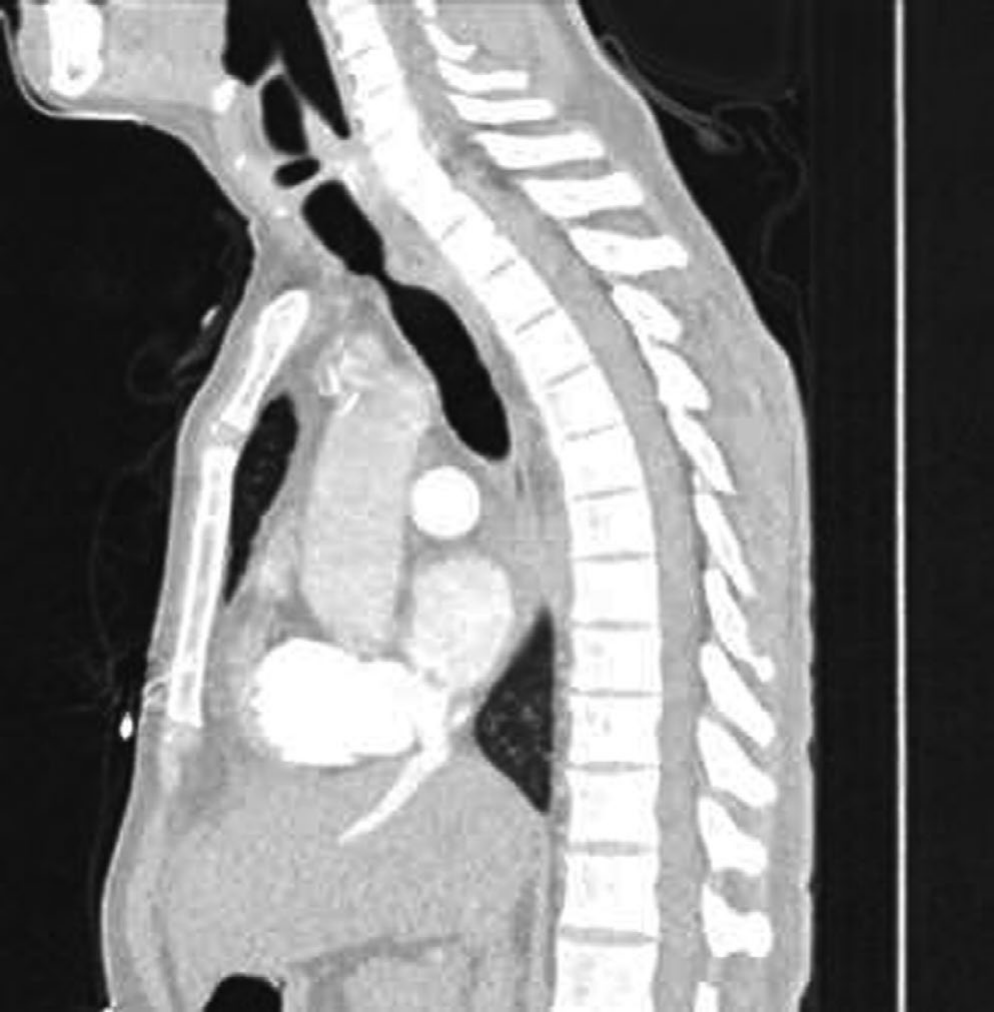
Sagittal section of the CT‐scan Chest showing the significant narrowing of the tracheal at the second thoracic vertebral level

A 55‐year‐old gentleman presented with fever, cough, and shortness of breath. He was diagnosed with ARDS secondary to severe COVID‐19 pneumonia. His clinical condition has deteriorated rapidly requiring intubation and mechanical ventilation for 13 days. A week following extubation, he became profoundly dyspneic with high oxygen requirements secondary to ventilator‐associated pneumonia (VAP) necessitating re‐intubation. Following 5 days of treatment, he was weaned successfully and later discharged for outpatient pulmonary rehabilitation. One month downstream, he re‐presented to the emergency department with stridor and worsening shortness of breath. Chest CT scan revealed critical tracheal narrowing at the level of the second thoracic vertebra (Figures 1, 2, 3). We performed a surgical tracheal segment resection with re‐anastomosis following multidisciplinary discussion and shared decision with the patient. After a short period of being supported by ECMO, he was decannulated and discharged home. At a follow‐up visit, he was doing well without stridor.

## CONFLICT OF INTEREST

The authors report no conflicts of interest in this work.

## AUTHORS' CONTRIBUTIONS

Dr. AME: served as corresponding author and involved in manuscript review and submission. Dr. MMA: served as co‐author and involved in manuscript writing, review, and finalization. Dr. MB, Dr. MH, Dr. MA, Dr. RM, and Dr. TR: involved in manuscript review.

## CONSENT FOR PUBLICATION

This case report does not contain any personal identifier of the patient, for example, name and photograph. It only includes radiological and pathological imaging. A written patient informed consent of patient information, diagnostic images, and publication was signed by the patient.

## Data Availability

The datasets used and/or analyzed during the current study are available from the corresponding author on reasonable request.

